# Supplementary material for: Purine Biosynthesis Pathways Are Required for Myogenesis in Xenopus laevis
Source: Cells. 2023 Sep 28;12(19):2379. doi: 10.3390/cells12192379 (PMC10571971; doi:10.3390/cells12192379)
Supplement: Supplementary file 1 [file cells-12-02379-s001.zip › cells-2628642-supplementary.pdf]

Supplementary Materials

Purine Biosynthesis Pathways Are Required for Embryonic Myogenesis in *Xenopus laevis*

Maëlle Duperray, Fanny Hardet, Elodie Henriet, Christelle Saint-Marc, Eric Boué-Grabot,
Bertrand Daignan-Fornier, Karine Massé and Benoît Pinson

Supplementary Figures

Figure S1. Conservation of purine biosynthesis pathways between the yeast *Saccharomyces cerevisiae* and *Xenopus laevis* p. 2
Figure S2. Sequence comparison between the *H. sapiens* (*Hs*) and *X. laevis* (*Xl*) adenylosuccinate lyase enzymes p. 3
Figure S3. Validation of the morpholinos translation interference by *in vitro* translation p. 4
Figure S4. Functional complementation of yeast purine *de novo* pathway mutants by the *X. laevis* ortholog genes p. 5
Figure S5. Functional complementation of yeast purine salvage knocked-out mutants by the *X. laevis* ortholog genes p. 6
Figure S6. Temporal expression profiles of purine pathway genes during embryogenesis p. 7
Figure S7. The expression profile was determined for each gene by *in situ* hybridization (ISH) using antisense probes p. 8
Figure S8. *In situ* hybridization using control sense probes p. 9
Figure S9. Severity of *adsl.L* knock-down-associated phenotypes is morpholino dose-dependent p. 10
Figure S10. The *ppat.L* and *ppat.S* genes are required for somites and hypaxial muscle formation in *X. laevis* p. 11
Figure S11. The *hprt1.L* gene is required for somitogenesis and hypaxial muscle formation in *X. laevis* p. 12
Figure S12. The *ppat.L*/*ppat.S* and *hprt1.L* genes are required for expression of the *tcf15* gene. p. 13
Figure S13. The *ppat.L*/*ppat.S* and *hprt1.L* genes are required for expression of the *mespa* gene. p. 14
Figure S14. Effect of *H. sapiens ADSL* and *X. laevis adsl.L* RNA* on *myod1* expression at stage 12.5 p. 15
Figure S15. Expression of the myogenic regulatory factor *myod1* gene in the paraxial mesoderm is strongly affected by knock-down of *ppat* and *hprt1.L* genes p. 16
Figure S16. Statistical analysis of the effects associated with *adsl.L* knock-down on *myogenin* expression in craniofacial muscles in late tailbud stage embryos p. 17
Figure S17. Statistical analysis of the effects consecutive to *adsl.L* knock-down on *myod1* expression in hypaxial muscles in late tailbud stage embryos p. 17

Supplementary Tables

Table S1: Yeast strains p. 18
Table S2: Plasmids used for functional complementation in yeast p. 19
Table S3: Plasmids used for mRNA synthesis p. 19
Table S4: Ribonucleotides probes used for *in situ* hybridization p. 20
Table S5: Oligonucleotides used for RT-PCR analyses p. 21
Table S6: Comparison of the purine biosynthesis pathways encoding genes and proteins between, *X. laevis, H. sapiens* and *X. tropicalis* p. 22-24
Table S7: Comparison of the purine biosynthesis pathways encoding genes and proteins between *X. laevis* and *S. cerevisiae* p. 24-27

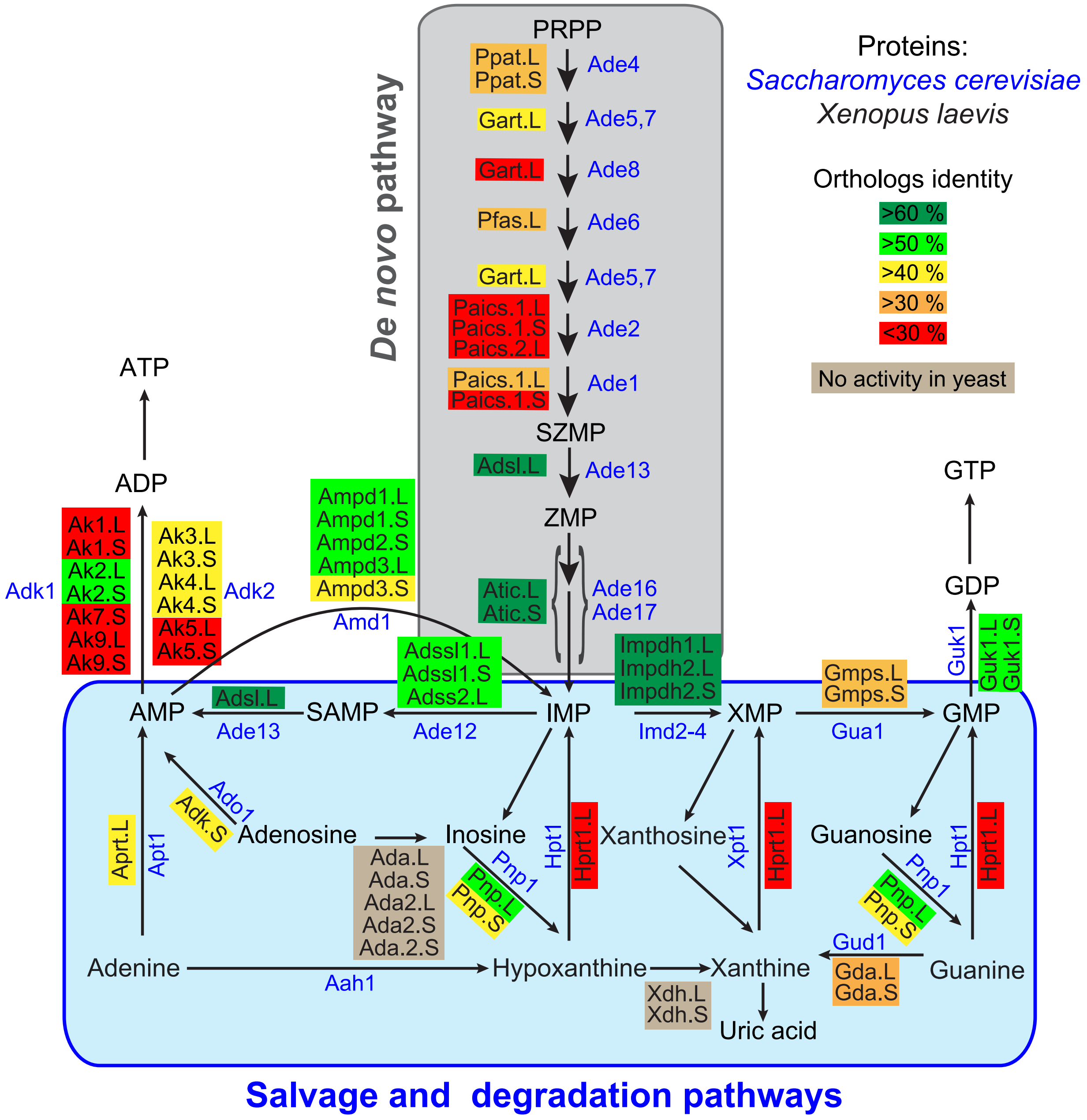

Figure S1. Conservation of purine biosynthesis pathways between the yeast *Saccharomyces cerevisiae* and *Xenopus laevis*. Features of the *X. laevis* and yeast enzymes are listed in Table S7. Abbreviations: AMP: adenosine monophosphate; GMP: guanosine monophosphate; IMP: Inosine monophosphate; PRPP: Phosphoribosyl pyrophosphate; SAMP: Succinyl-AMP; SZMP: Succinyl Amino Imidazole Carboxamide Ribonucleotide monophosphate; XMP: Xanthosine monophosphate; ZMP: Amino Imidazole CarboxAmide Ribonucleotide monophosphate.

```
ADSL    Hs     1-MAAGGDHGSPDS--------------------YRSPLASRYASPEMCFVFSDRYKFRTWRQLWLWLAEAEQTLGLPITDEQIQEMKSNLENI-72
               1-M..G....S.DS--------------------YRSPL.SRYAS.EM.F.FSD..KF.TWR.LWLWLA.AE..LGLPIT.EQIQEM..NLENI
Adsl.L  Xl     1-ME-GSSGLSMDSNTRITGSVSPLTPGPEEVMRYRSPLVSRYASREMAFNFSDSKKFQTWRRLWLWLAQAERSLGLPITEEQIQEMEANLENI-91

ADSL    Hs    73-DFKMAAEEEKRLRHDVMAHVHTFGHCCPKAAGIIHLGATSCYVGDNTDLIILRNALDLLLPKLARVISRLADFAKERASLPTLGFTHFQPAQ-164
                 DFKMAAEEEKRLRHDVMAHVHTF.HCCPKAA..IHLGATSCYVGDNTDLI.LR...DLLLPKLARV..RLADFA...A..PTLGFTH.QPAQ
Adsl.L  Xl    92-DFKMAAEEEKRLRHDVMAHVHTFAHCCPKAAPVIHLGATSCYVGDNTDLIVLRDGFDLLLPKLARVLNRLADFAEKYAEMPTLGFTHYQPAQ-183

ADSL    Hs   165-LTTVGKRCCLWIQDLCMDLQNLKRVRDDLRFRGVKGTTGTQASFLQLFEGDDHKVEQLDKMVTEKAGFKRAFIITGQTYTRKVDIEVLSVLA-256
                 LTTVGKR.CLW.QDLCMDL..L.R.R..LRFRGVKGTTGTQASFLQLF.GD..KVE.LD.MVT..AGFKRA.I.TGQTY.RKVD.EV.SVLA
Adsl.L  Xl   184-LTTVGKRACLWLQDLCMDLRNLERARNELRFRGVKGTTGTQASFLQLFDGDHDKVEELDRMVTSMAGFKRAYIVTGQTYSRKVDVEVVSVLA-275

ADSL    Hs   257-SLGASVHKICTDIRLLANLKEMEEPFEKQQIGSSAMPYKRNPMRSERCCSLARHLMTLVMDPLQTASVQWFERTLDDSANRRICLAEAFLTA-348
                 SLGA.VHKICTDIRLLANLKE.EEPFEK.QIGSSAMPYKRNPMRSERCCSLARHLMTL.M.PLQTASVQWFERTLDDSANRRICLAEAFLTA
Adsl.L  Xl   276-SLGATVHKICTDIRLLANLKELEEPFEKEQIGSSAMPYKRNPMRSERCCSLARHLMTLIMNPLQTASVQWFERTLDDSANRRICLAEAFLTA-367

ADSL    Hs   349-DTILNTLQNISEGLVVYPKVIERRIRQELPFMATENIIMAMVKAGGSRQDCHEKIRVLSQQAASVVKQEGGDNDLIERIQVDAYFSPIHSQL-440
                 D IL.TLQNISEGLVVYPKVIERRIRQELPFMATENIIMAMVK.GG.RQDCHE.IRVLSQQA..VVKQEGGDNDLI.RIQ.D.YF.P.HA.H
Adsl.L  Xl   368-DIILSTLQNISEGLVVYPKVIERRIRQELPFMATENIIMAMVKNGGNRQDCHERIRVLSQQAGAVVKQEGGDNDLIFRIQSDSYFAPIHAHL-459

ADSL    Hs   441-FSPIHSQLDHLLDPSSFTGRASQQVQRFLEEEVYPLLKPYESVMKVKAELCL-484
                 F.PIH..L..LLDP.SF.GRA.QQV..FL.EEV.PLL.PY.S.M.VK.EL.L
Adsl.L  Xl   460-FAPIHAHLEQLLDPKSFIGRAPQQVLKFLKEEVIPLLSPYQSKMDVKMELEL-503
```

Figure S2. Sequence comparison between the *H. sapiens* (*Hs*) and *X. laevis* (*Xl*) adenylosuccinate lyase enzymes. Conserved amino acids are indicated in bold. Red and orange boxes point to residues required for catalysis and yellow and orange boxes to mutated residues found in *ADSL*-deficient patients.

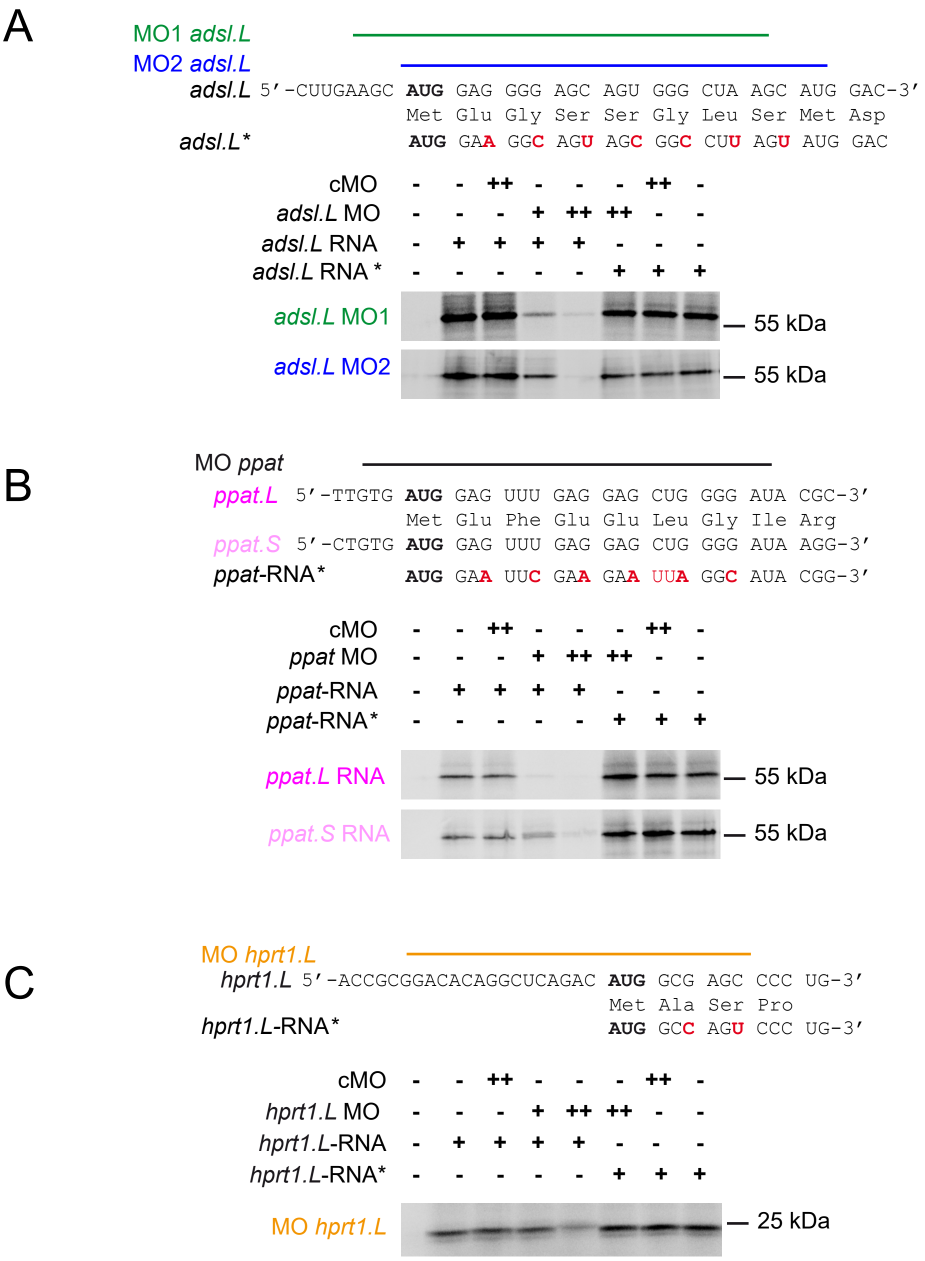

Figure S3. Validation of the morpholinos translation interference by *in vitro* translation. The *adsl.L* (**A**), *ppat.L/ppat.S* (**B**) and *hprt1.L* (**C**) RNAs were produced using the *mMessage mMachine SP6* kit (ambion) according to the supplier procedure. *In vitro* translation was then performed by using the reticulocyte rabbit lysate system kit (Promega) in 25 µl reaction mix containing 500 ng of indicated RNA, 0.5 µl of the amino acid mix w/o Met (1 mM), 2 µl of [^{35}S]-methionine (1,200 Ci/mmol; 10 mCi/ml), 17.5 µl of the reticulocytes lysate, 0.5 µL of RNAse inhibitor (40 u/µl; Promega) and in the presence or the absence of 40 (+) or 400 (++) ng of indicated Morpholino (MO). Proteins were separated by SDS-PAGE and radiolabeled proteins were detected by phosphorimaging (Typhoon biomolecular imager, Amersham). RNA* refers to mutated RNAs whose translation is not affected by the gene-specific MOs.

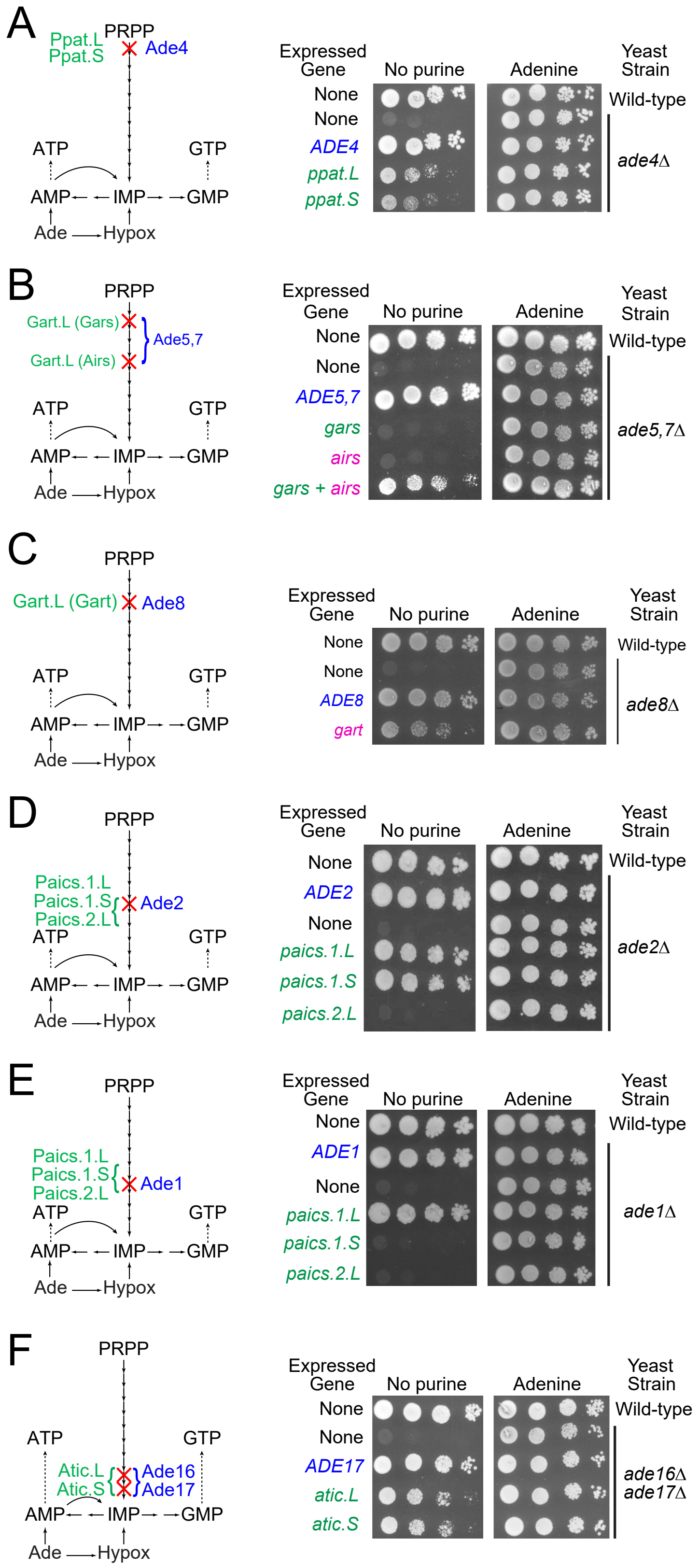

Figure S4. Functional complementation of yeast purine *de novo* pathway mutants by the *X. laevis* ortholog genes. Red crosses indicate the purine pathway steps knocked-out in each yeast mutant. *S. cerevisiae*, *X. laevis* and *X. tropicalis* protein or gene names are written in blue, green and pink, respectively. For Airs and Gart activities, functional complementation was performed by expressing the *X. tropicalis* corresponding open reading frames. Abbreviations: Ade: adenine; Airs; aminoimidazole ribonucleotide synthetase activity; Gars: glycinamide ribonucleotide synthetase activity; Gart: glycinamide ribonucleotide transformylase activity; Hypox: hypoxanthine; IMP: Inosine monophosphate; PRPP: Phosphorybosyl pyrophosphate. Yeast mutants were transformed with plasmids allowing expression of the indicated yeast (blue) or *X. laevis* (green) or *X. tropicalis* (pink) genes or with the empty vector (None). Transformants were serial (1/10) diluted and spotted on SDcasaW medium supplemented or not (no purine) with adenine as sole external purine source. Plates were imaged after 2 (**F**), 4 (**A**, **D-E**) or 7 days (**B-C**) at either 30°C (**A**, **D-F**) or 37°C (**B-C**).

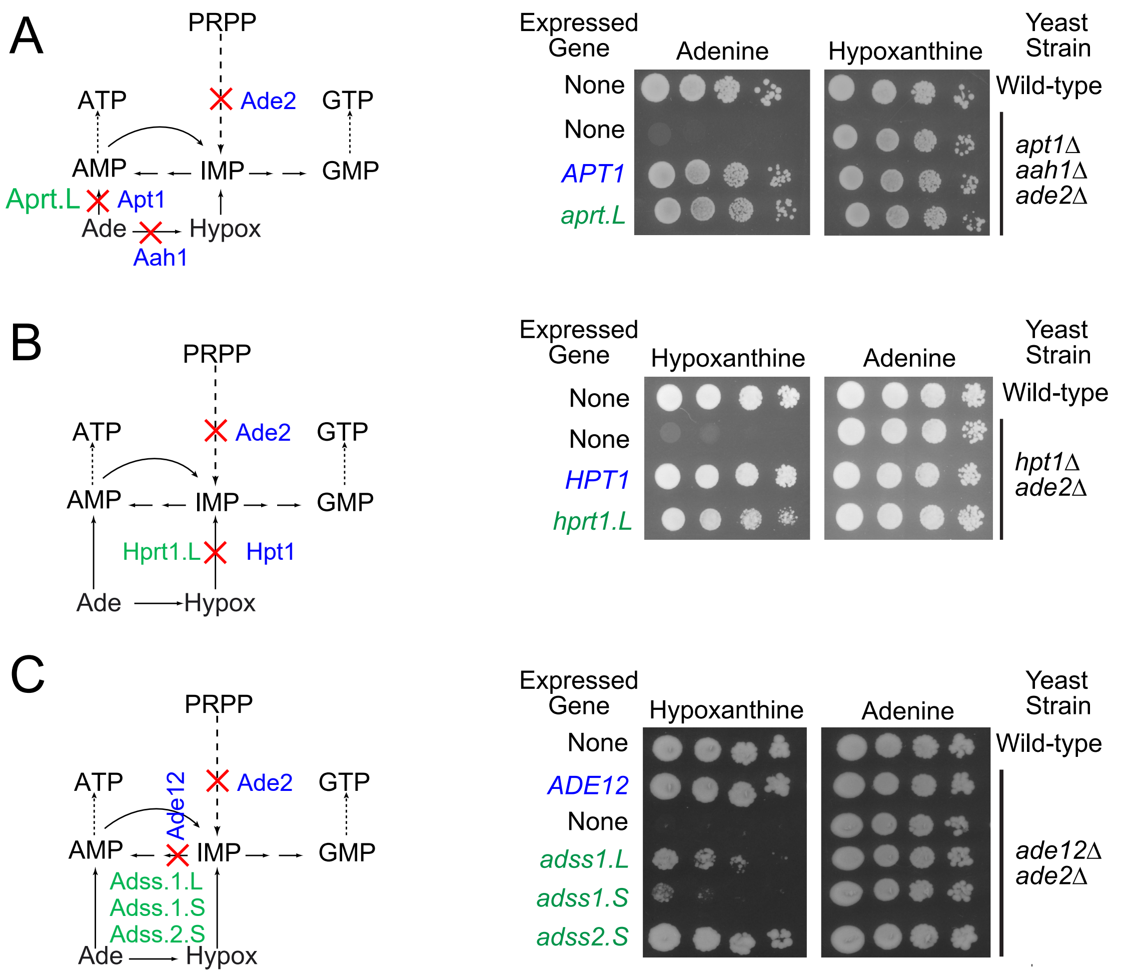

Figure S5. Functional complementation of yeast purine salvage knocked-out mutants by the *X. laevis* ortholog genes. The vertical dashed line symbolizes the *de novo* purine pathway. Red crosses are used to indicate the purine pathway steps absent in each yeast knock-out mutant. *S. cerevisiae* and *X. laevis* protein or gene names are written in blue and green, respectively. Ade: Adenine; Hypox: Hypoxanthine; IMP: Inosine monophosphate; PRPP: 5-phosphorybosyl-pyrophosphate. Wild-type and mutant yeast strains were transformed with plasmids allowing expression of the indicated *S. cerevisiae* (blue) or *X. laevis* (green) genes or with the empty vector (None). Transformants were serial diluted (1/10) and spotted on SDcasaW medium supplemented with either adenine or hypoxanthine as sole external purine source. Plates were imaged after 2 days at 30°C.

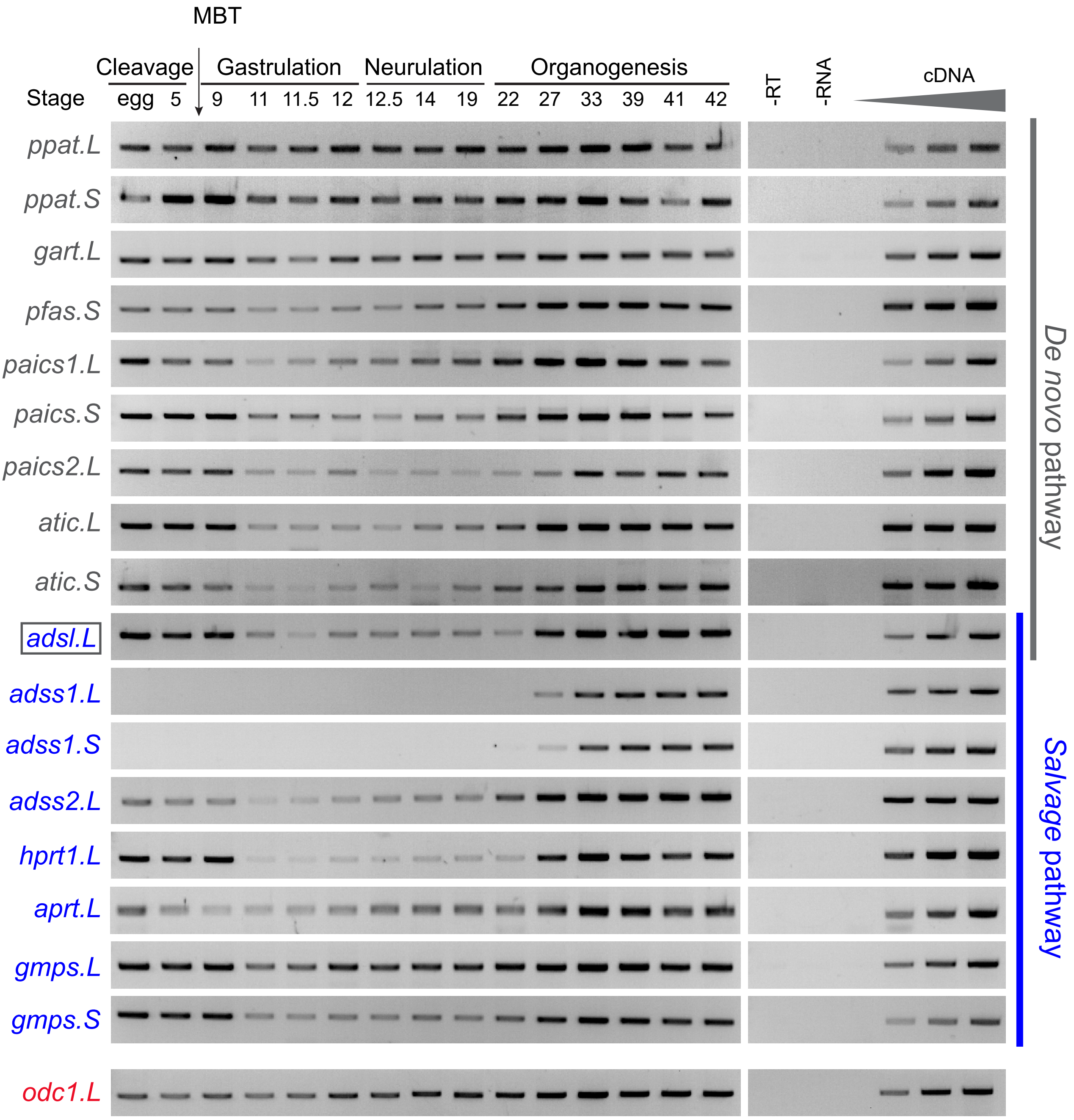

Figure S6. Temporal expression profiles of purine pathway genes during embryogenesis. The expression profile was determined for each gene by RT-PCR from cDNA of fertilized egg and whole embryo at different stages covering the different phases of *X. laevis* embryogenesis. Negative controls were performed in the absence of either reverse transcriptase (-RT) or total RNA extract (-RNA). The linearity was performed with doubling dilutions of cDNA. A loading control was done using *odc1.L* gene (in red). Names corresponding to genes encoding enzymes of the purine *de novo* and salvage pathways are written in grey and blue, respectively. The *adsl.L* gene encoding the adenylosuccinate lyase involved in both purine pathways is boxed in grey. Experiments were done at least twice ($n \geq 2$) on embryos obtained from *in vitro* fertilization of oocytes from two different females (N=2). Sense and antisense primers are described in Table S5. MBT: mid blastula transition.

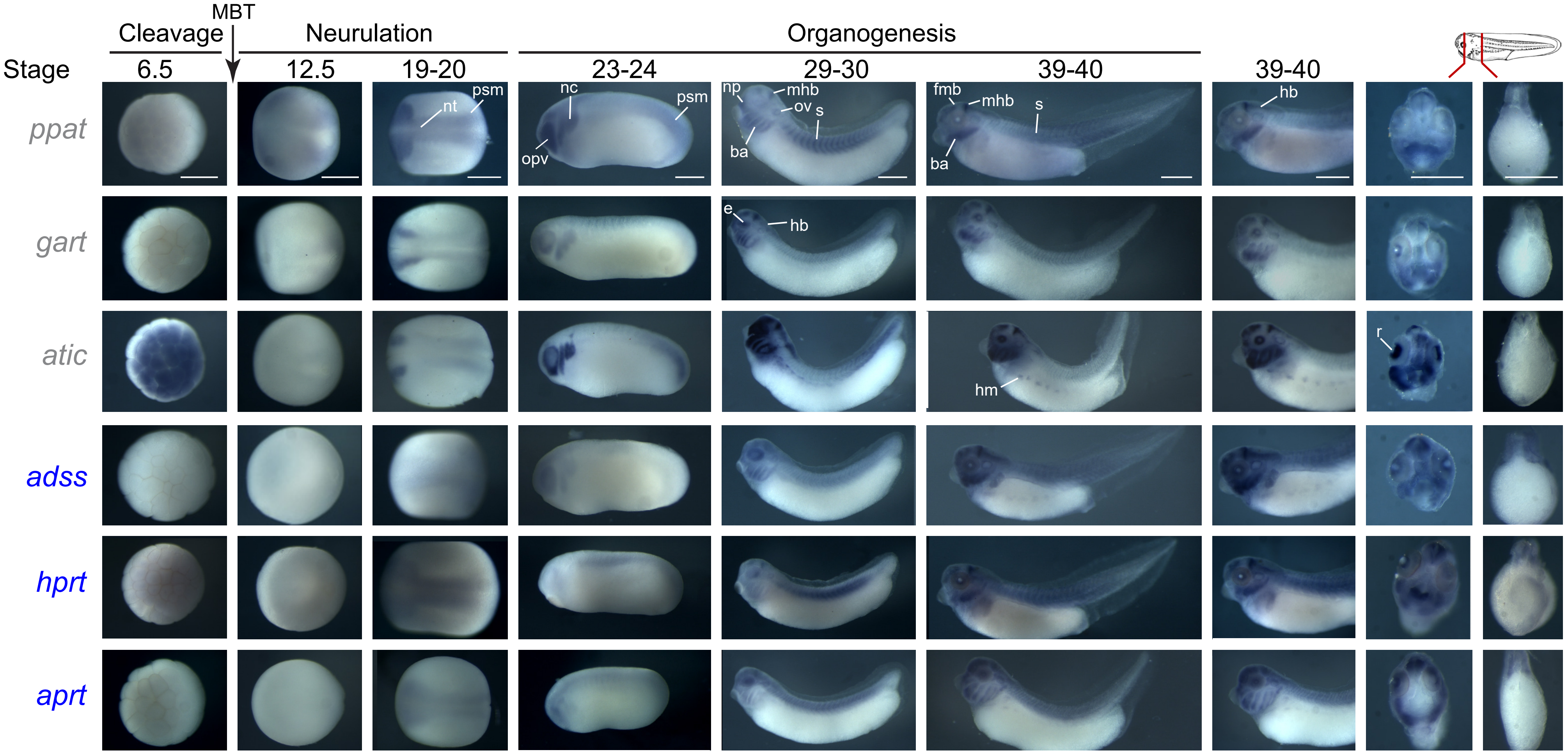

Figure S7. The expression profile was determined for each gene by *in situ* hybridization using antisense riboprobes (Table S4). No unspecific staining was detected with the control sense probes (Figure S8). MBT: Mid blastula transition. Grey and blue gene name colors refer to *de novo* and salvage purine pathways, respectively. Representative embryos were photographed. Stage 6.5: animal pole view, later stages: lateral views, with dorsal is up and anterior is left. Transverse section: dorsal is up. Abbreviations: ba, branchial arches; e, eye; fmb, forebrain-midbrain boundary; hb, hindbrain; hm, hypaxial muscles; l, lens; mhb, midbrain-hindbrain boundary; n: nasal placode; nc, neural crest; np, neural plate; nt, neural tube; opv, optical vesicle; ov, otic vesicle; psm, presomitic mesoderm; r, retina; s, somites. Bars: 0.5 mm.

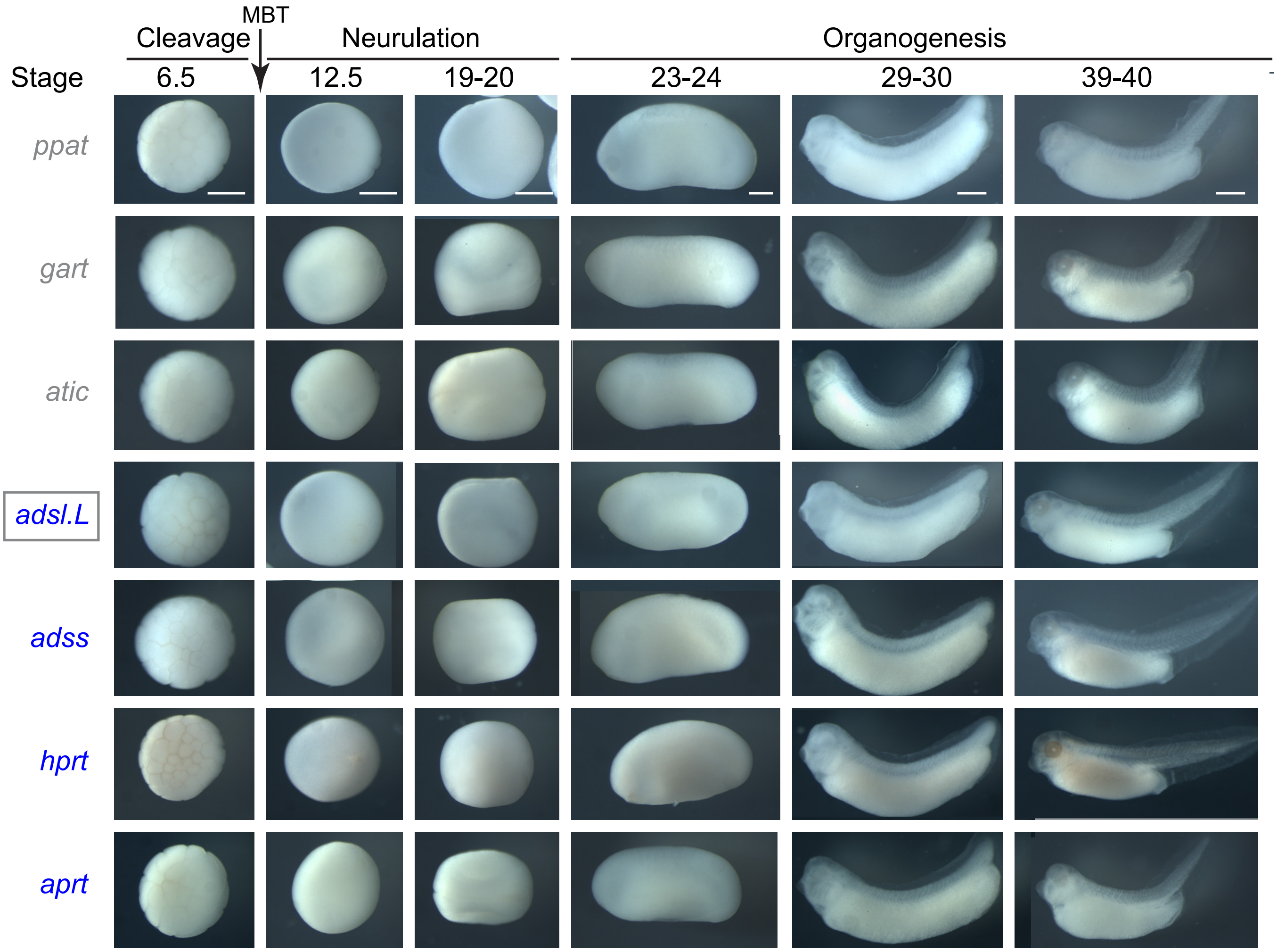

Figure S8. *In situ* hybridization using control sense riboprobes. *In situ* hybridization sense probes are described in Table S4. MBT: Mid blastula transition. Grey and blue gene name colors refer to *de novo* and salvage purine pathways, respectively. Representative embryos were photographed. Stage 6.5: animal pole view, later stages: lateral views, with dorsal is up and anterior is left. Bars: 0.5 mm.

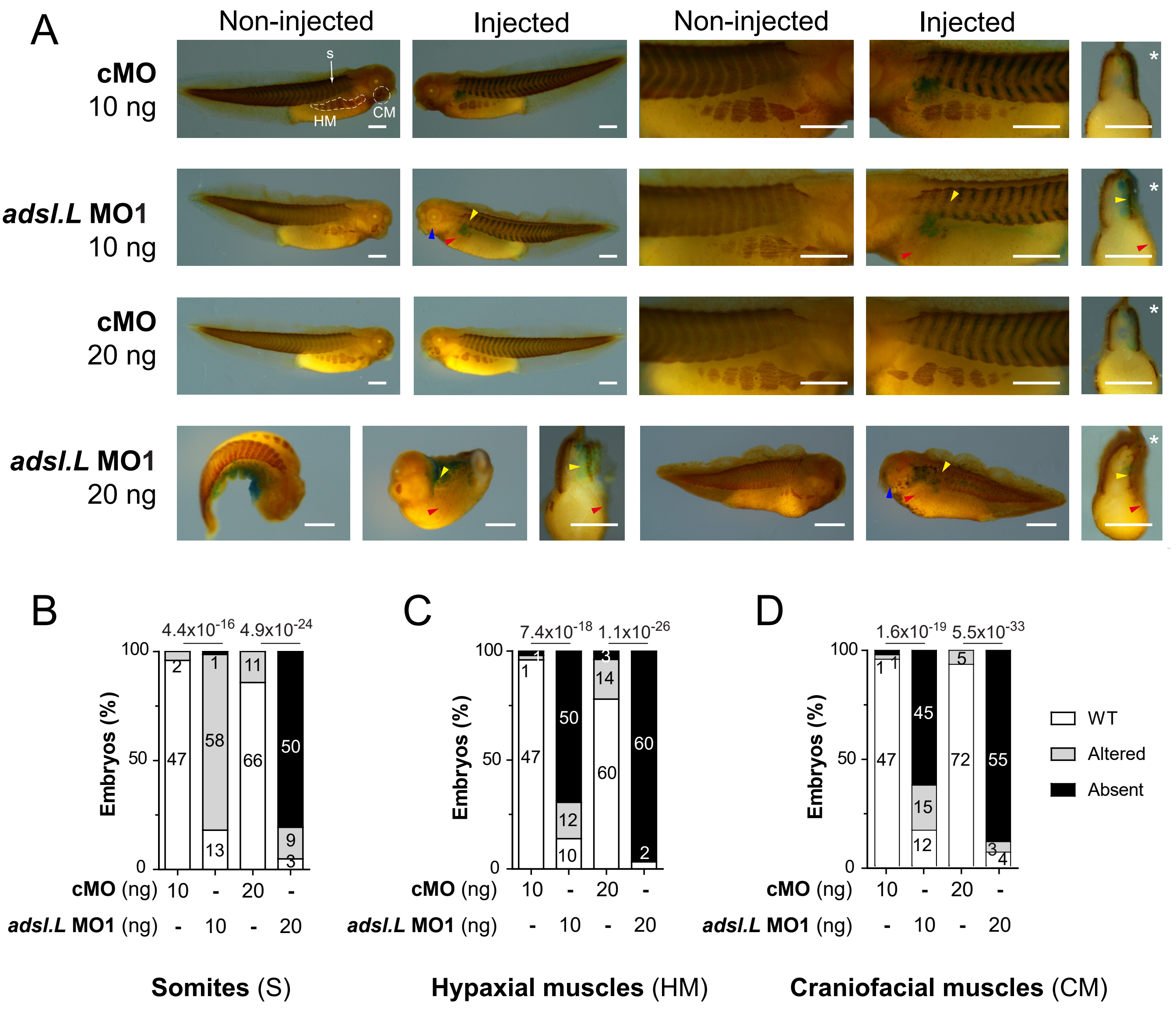

Figure S9. Severity of *adsl.L* knock-down-associated phenotypes is morpholino dose-dependent. 12-101 immunolabelling revealed a strong alteration of myogenesis and somitogenesis (**A**-**B**), hypaxial (**A**, **C**) and craniofacial (**A**, **D**) muscle formation in *adsl.L* morphant embryos. Representative images (**A**), quantification (embryo numbers in bars) and statistics of somite (**B**), hypaxial muscle (**C**) and craniofacial muscle (quadratoangularis + levator mandibularis longus) (**D**) phenotypes at tadpole stage. Yellow, blue and red arrowheads points to regions of somitic (S), craniofacial (CM) and hypaxial (HM) muscle defaults, respectively. Asterisks show injected side. Bars: 0.5 mm. Numbers above the bars in the histograms correspond to p-values.

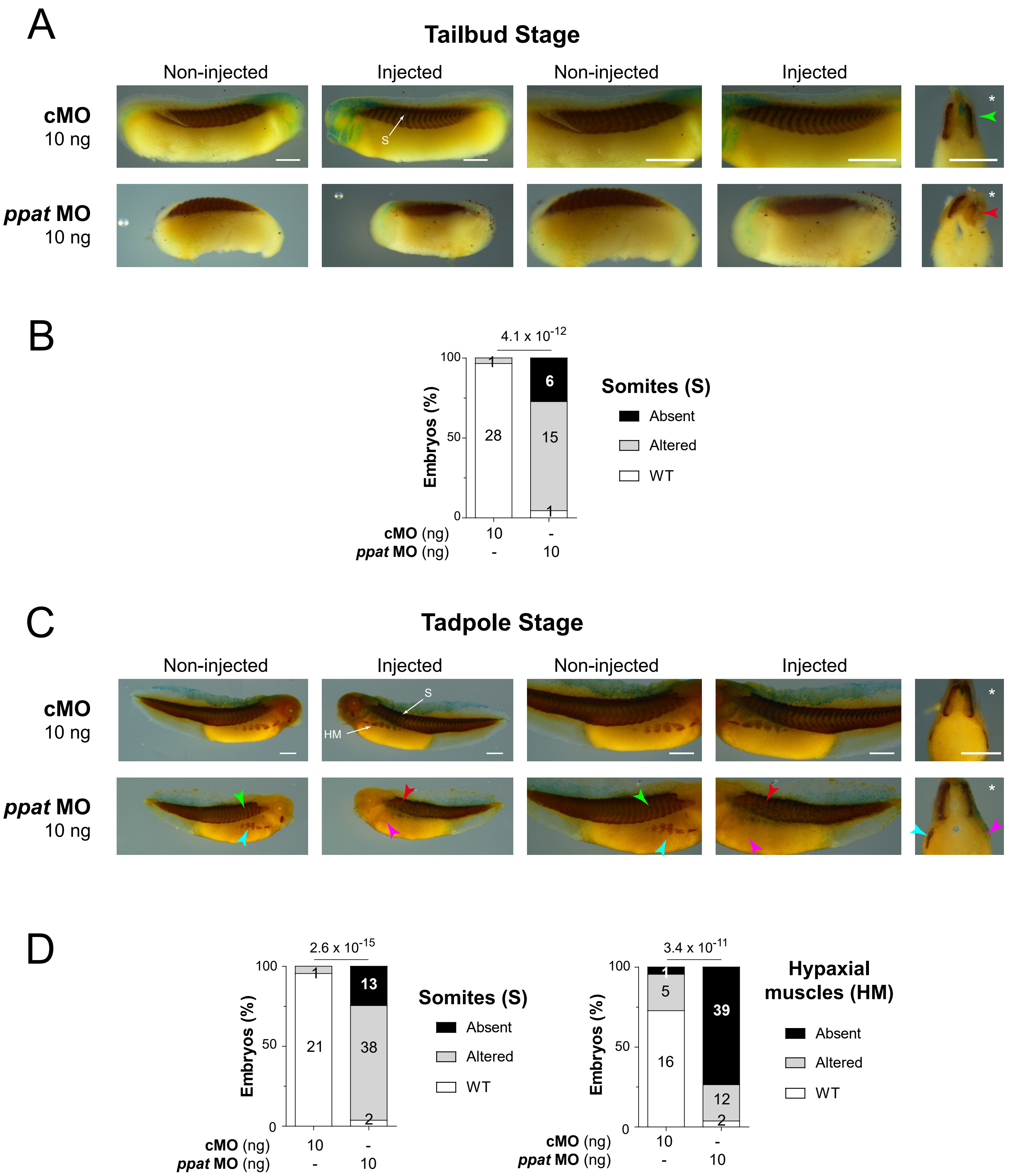

Figure S10. The *ppat.L* and *ppat.S* genes are required for somite, myotome and hypaxial muscle formation in *Xenopus laevis*. (**A-D**) A strong muscle alteration in *ppat* knock-down embryos is revealed by 12-101 immunolabelling. Representative images (**A, C**), quantification (embryo numbers in bars) and statistics (**B, D**) of somite and hypaxial muscle phenotypes at tailbud (**A-B**) and tadpole (**C-D**) stages. Green and red arrowheads point to normal v-shaped and altered somites, respectively; blue and pink arrowheads show normal and reduced 12/101 positive hypaxial muscle area, respectively. Injected side is indicated by asterisks. S: somites; HM: Hypaxial muscles. Bars: 0.5 mm. Numbers above the bars in the histograms correspond to p-values.

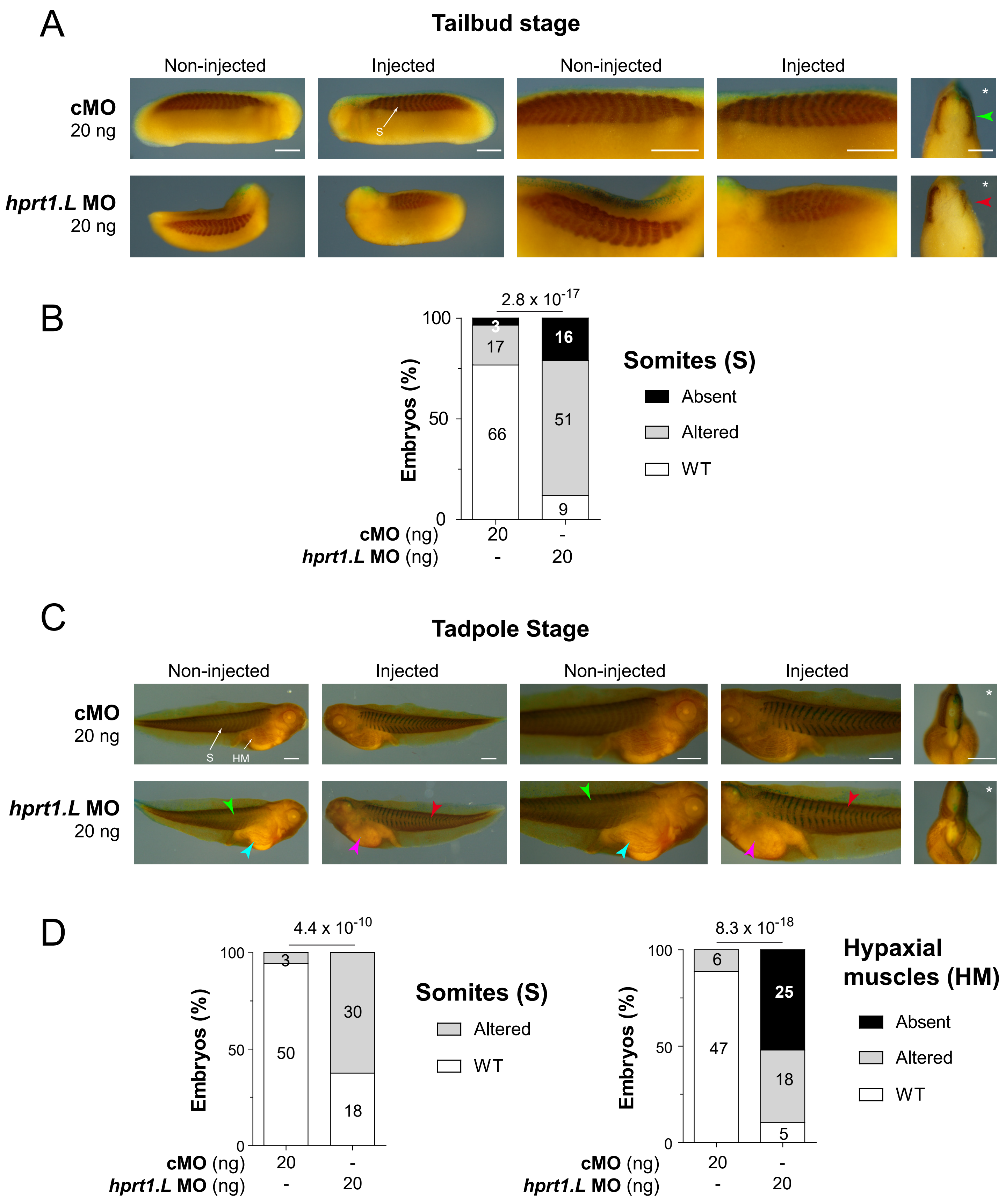

Figure S11. The *hprt1.L* gene is required for somite, myotome and hypaxial muscle formation in *Xenopus laevis*. (**A-D**) A strong muscle alteration is revealed by 12-101 immunolabelling in *hprt1.L* knock-down embryos. Representative images (**A, C**), quantification (embryo numbers in bars) and statistics (**B**, **D**) of somite and hypaxial muscle phenotypes at tailbud (**A-B**) and tadpole (**C-D**) stages. Green and red arrowheads point to normal v-shaped and altered somites, respectively; blue and pink arrowheads show normal and reduced 12/101 positive hypaxial muscle area, respectively. Injected side is indicated by asterisks. S: somites; HM: Hypaxial muscles. Bars: 0.5 mm. Numbers above the bars in the histograms correspond to p-values.

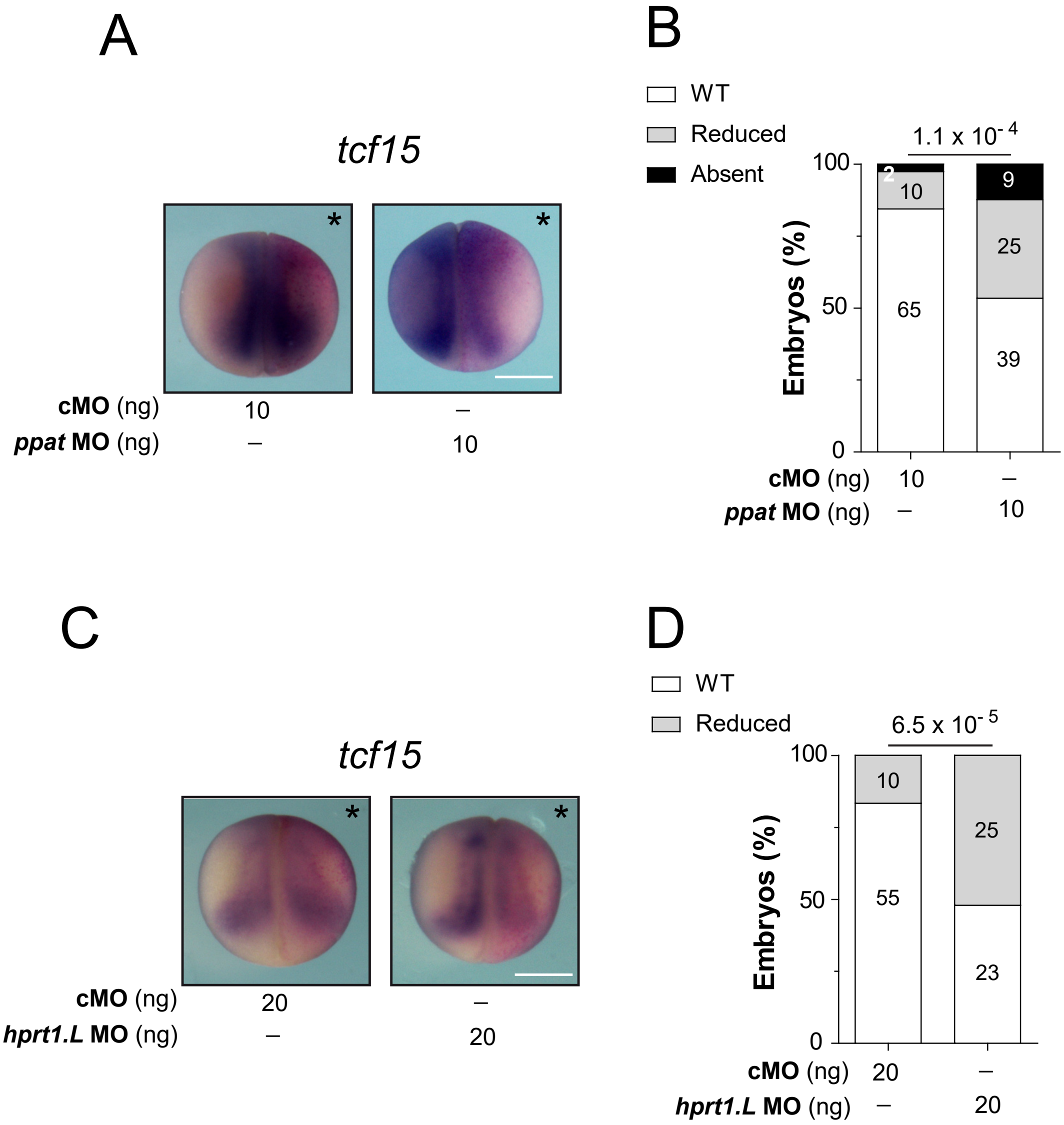

Figure S12. The *ppat.L*/*ppat.S* and *hprt1.L* genes are required for expression of *tcf15* gene. Expression of *tcf15* RNA was monitored by *in situ* hybridization at late neurula stage (stage 17-19) on embryos injected with the control morpholino (cMO) or the specific MO targeting either *ppat* or *hprt1.L* genes. Representatives images are presented for *ppat* (**A**) and *hprt1.L* (**C**) genes. Quantification (numbers in bars) and statistics of the *tcf15* expression phenotypes are presented in (**B**, **D**). Injected side is indicated by asterisks. Bars: 0.5 mm. Numbers above the bars in the histograms correspond to p-values.

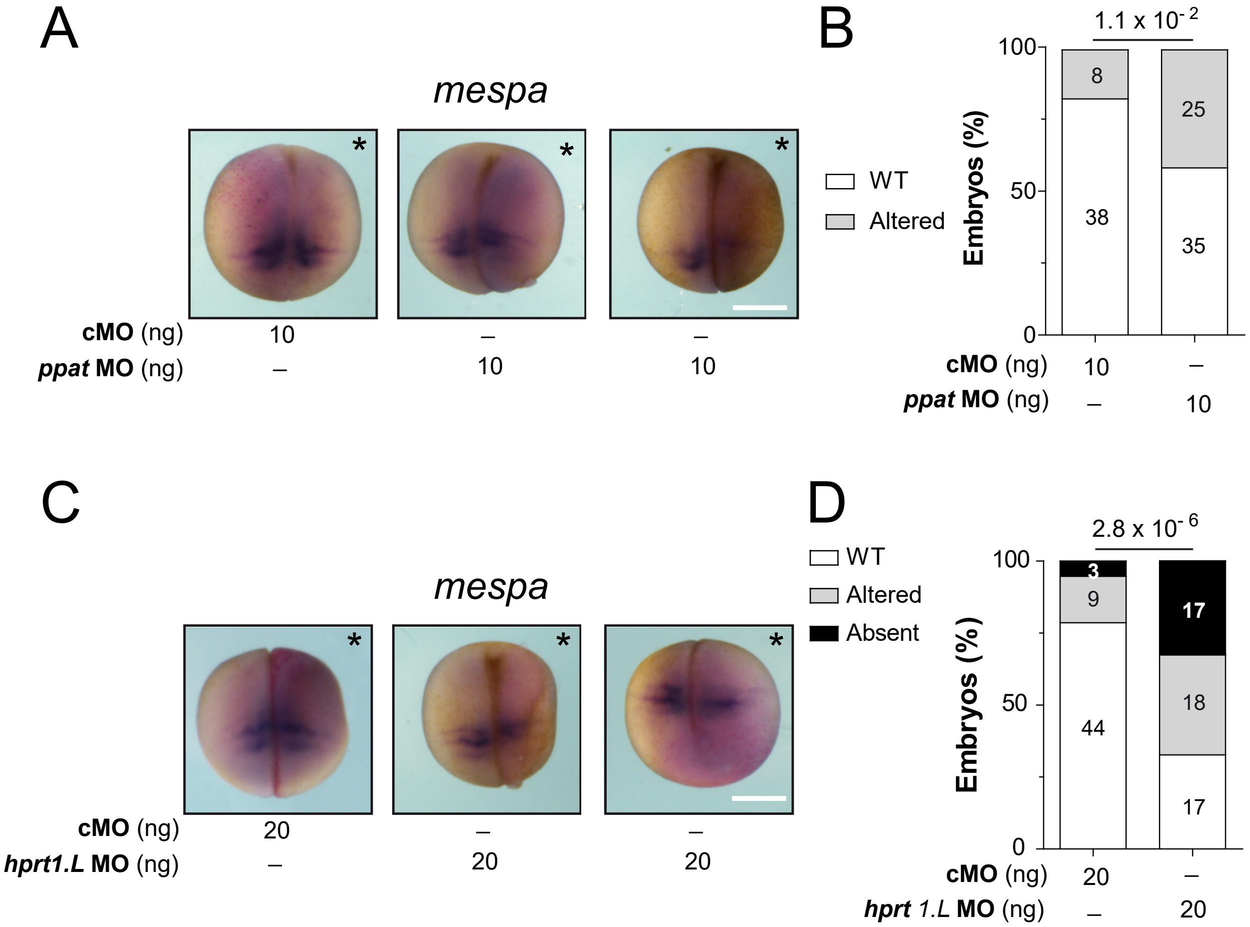

Figure S13. The *ppat.L*/*ppat.S* and *hprt1.L*genes are required for *mespa* gene expression. Expression of *mespa* RNA was monitored by *in situ* hybridization at late neurula stage (stage 17-19) on embryos injected with the control morpholino (cMO) or the specific MO targeting either *ppat* or *hprt1.L* genes. Representatives images are presented for *ppat* (**A**) and *hprt1.L* (**C**) genes, respectively. Quantification (numbers in bars) and statistics of the *mespa* expression phenotypes are presented in (**B**, **D**). Injected side is indicated by asterisks. Bars: 0.5 mm. Numbers above the bars in the histograms correspond to p-values.

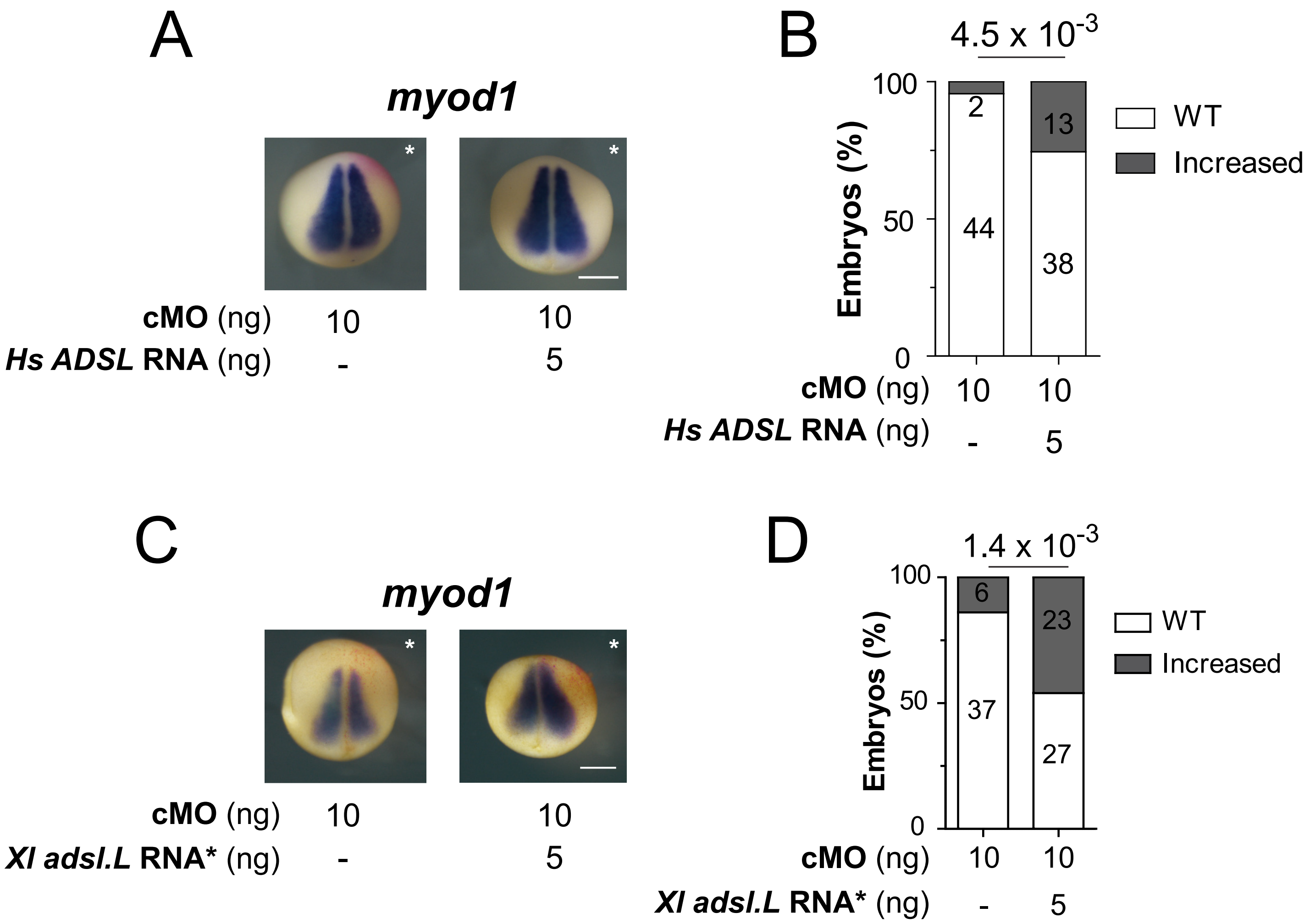

Figure S14. Effect of *H. sapiens ADSL* and *X. laevis adsl.L* RNA* on *myod1* expression at stage 12.5. (**A, C**) Expression of *myod1* was monitored by *in situ* hybridization at stage 12.5 on embryos injected with the control morpholino (cMO) and co-injected or not (-) with either the *H. sapiens ADSL* RNA (**A**) or the *X. laevis* MO non-targeted *adsl.L* RNA* (**C**), (**B, D**) Quantification (numbers in bars) and statistics of the *myod1* expression phenotypes are presented in (**A**, **C**). Injected side is indicated by asterisks. Bars: 0.5 mm. Numbers above the bars in the histograms correspond to p-values.

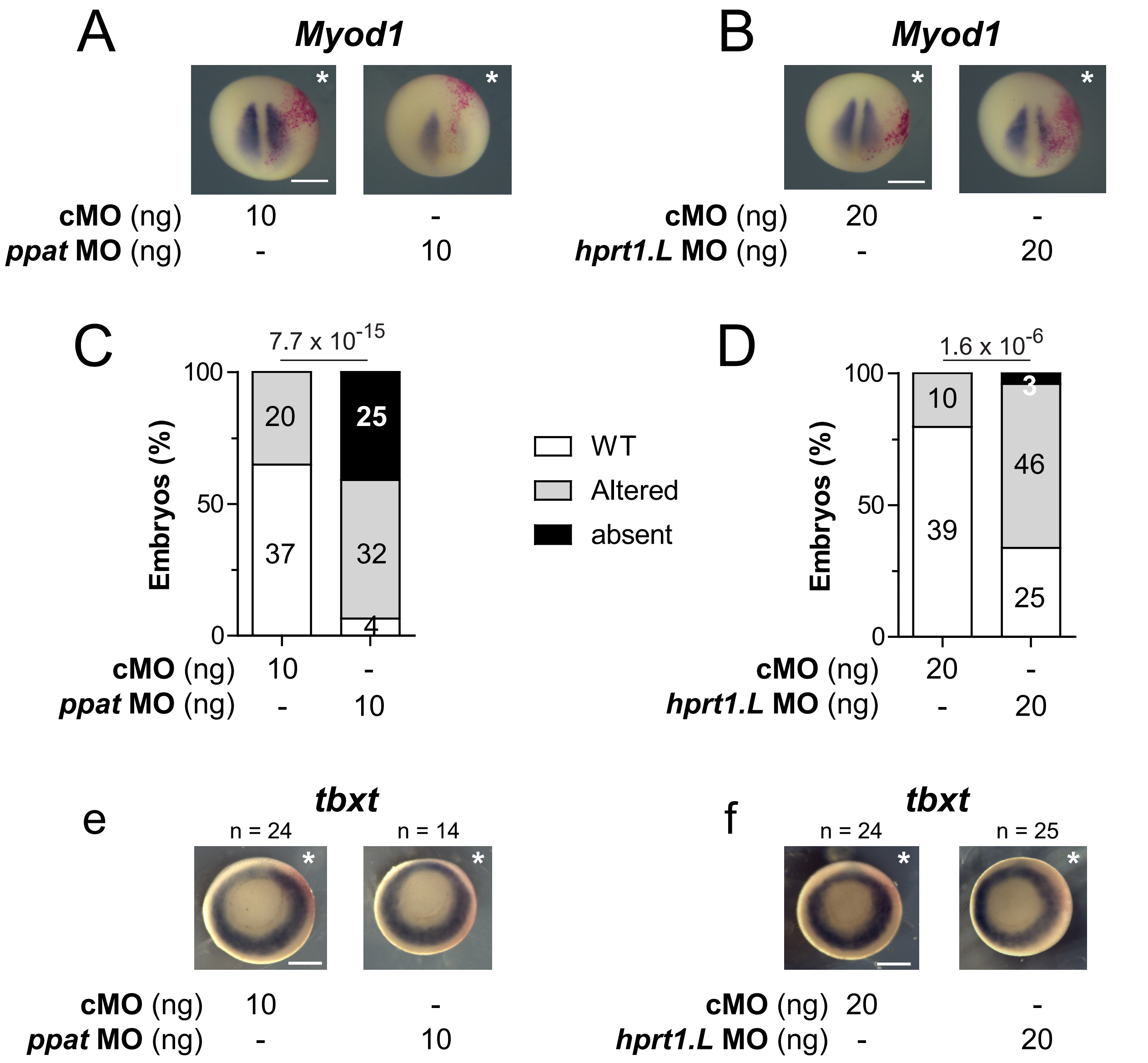

Figure S15. Expression of the myogenic regulatory factor *myod1* gene in the paraxial mesoderm is strongly affected by knock-down of *ppat* and *hprt1.L* genes. (**A-B**) Representative images of *myod1* expression alteration by the knock-down of either *ppat.L*/*ppat.S* (**A**) or *hprt1.L* (**B**) genes at stage 12.5, (**C-D**) Quantification (embryo numbers in bars) and statistics of the *myod1* expression phenotypes presented in (**A-B**). (**E-F**) Knock-down of both *ppat.L*/*ppat.S* (**E**) or *hprt1.L* (**F**) genes has no significant effect on general mesoderm formation, as revealed by the pan-mesoderm *tbxt* (*xbra*) expression pattern at stage 11. Injected side is indicated by asterisks. Bars: 0.5 mm. Numbers above the bars in the histograms correspond to p-values.

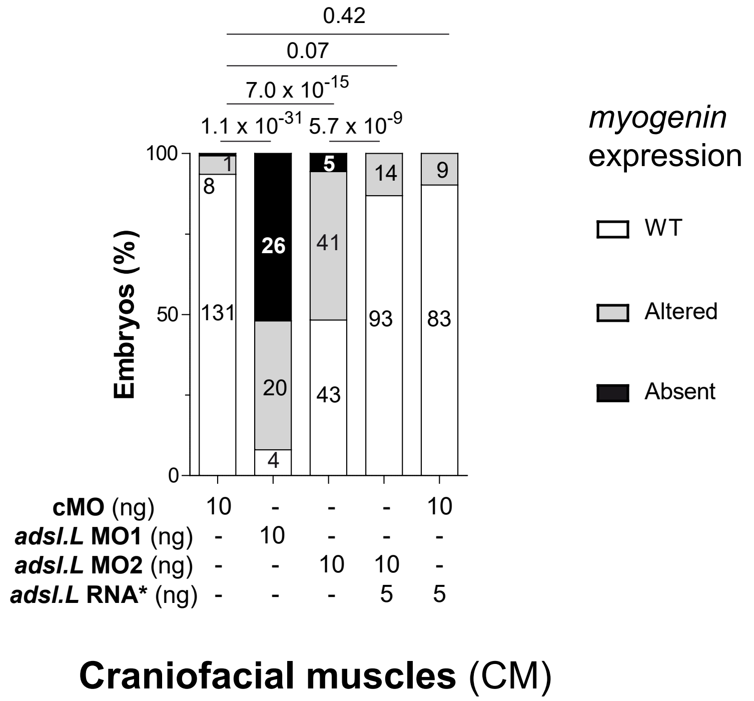

Figure S16. Statistical analysis of the effects associated with *adsl.L* knock-down on *myogenin* expression in craniofacial muscles in late tailbud stage embryos. Quantification (numbers in bars) and statistics of the *myogenin* expression phenotypes presented in Figure 6A. Numbers above the bars in the histograms correspond to p-values.

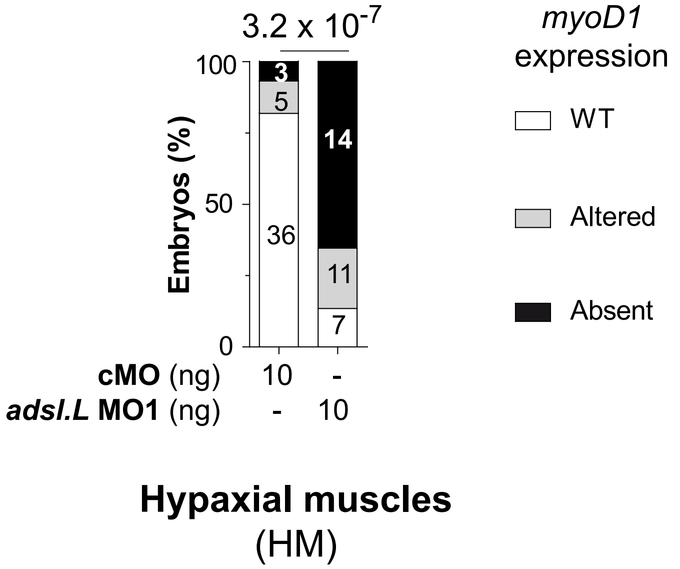

Figure S17. Statistical analysis of the effects consecutive to *adsl.L* knock-down on *myod1* expression in hypaxial muscles in late tailbud stage embryos. Quantification (numbers in bars) and statistics of the *myod1* expression phenotypes presented in Figure 5C. Numbers above the bars in the histograms correspond to p-values.

Table S1: Yeast strains

Strain	Genotype	Reference
BY4741	*Matα ura3Δ leu2Δ his3Δ lys2Δ*	Euroscarf
BY4742	*Mat***a** *ura3Δ leu2Δ his3Δ met15Δ*	Euroscarf
DS1-2B/1	*Matα ura3Δ ade2 apt1 aah1*	R. Woods
Y1036	*Matα ura3Δ leu2Δ his3Δ lys2Δ ade1::kanMX4*	Lab collection
Y1057	*Matα ura3Δ leu2Δ his3Δ lys2Δ ade4::kanMX4*	Lab collection
Y1059	*Matα ura3Δ leu2Δ his3Δ lys2Δ ade5,7::kanMX4*	Lab collection
Y1063	*Matα ura3Δ leu2Δ his3Δ lys2Δ ade8::kanMX4*	Lab collection
Y1095	*Matα ura3Δ leu2Δ his3Δ lys2Δ ade16::kanMX4 ade17::kanM*[illegible]	Lab collection
Y1133	*Mat*a *ura3Δ leu2Δ his3Δ lys2Δ ade8::kanMX4*	Lab collection
Y3574	*Matα ura3Δ leu2Δ his3Δ ade1::kanMX4 ade13::kanMX4*	Lab collection
Y8093	*Matα ura3Δ leu2Δ his Δ ade2::kanMX4 hpt1::kanMX4*	Lab collection
Y11114	*Matα ura3Δ leu2Δ his3Δ lys2Δ ade2::KanMX4 ade12::HIS3*	Lab collection

Table S2: Plasmids used for functional complementation in *S. cerevisiae*

Plasmid	I.M.A.G.E Clone used for amplification	Characteristics	Reference
pCM189	N/A	*CEN ARS URA3 tet-OFF promoter*	Gari 1997[a]
p4930	IRBHp990G1167D	*CEN ARS URA3 tet-atic.L. (X. laevis)*	This study
p4933	IRBHp990H0610D	*CEN ARS URA3 tet-adsl.L. (X. laevis)*	This study
p5153	IRAKp961G14156Q	*CEN ARS URA3 tet-atic.S. (X. laevis)*	This study
p5255	IRBHp990F0459D	*CEN ARS URA3 tet-paics.1.L. (X. laevis)*	This study
p5257	IRBHp990G071D	*CEN ARS URA3 tet-paics.1.S. (X. laevis)*	This study
p5303	IRBHp990C0135D	*CEN ARS URA3 tet-adss.1.S. (X. laevis)*	This study
p5304	IRBHp990B1280D	*CEN ARS URA3 tet-adss.1.L. (X. laevis)*	This study
P5318	IRBH990B02030D	*CEN ARS LEU2 tet-hprt.L (X. laevis)*	This study
p5321	IRAKp961E17253Q	*CEN ARS URA3 tet-aprt.L (X. laevis)*	This study
p5551	IRBHp990H1017D	*CEN ARS URA3 tet-ppat.L.(X. laevis)*	This study
p5400	IMAGp998O0914583Q	*CEN ARS URA3 tet-ppat.S (X. laevis)*	This study
p5550	IRBH990B02030D	*CEN ARS LEU2 tet-hprt.S (X. laevis)*	This study
p5697	IRAKp961P06157Q	*CEN ARS URA3 tet-paics.2 (X. laevis)*	This study
p5740	IMAGp998B1011965Q	*CEN ARS URA3 tet-gart (X. tropicalis)*	This study
P5744	IMAGp998B1011965Q	*CEN ARS URA3 tet-gars (X. tropicalis)*	This study
P5269	IRAKp961C16299Q	*CEN ARS LEU2 tet-airs (X. laevis)*	This study

[a] E Garí, L Piedrafita, M Aldea, E Herrero A set of vectors with a tetracycline-regulatable promoter system for modulated gene expression in Saccharomyces cerevisiae **1997** *Yeast* 13(9):837-48. DOI: 10.1002/(SICI)1097-0061(199707)13:9<837::AID-YEA145>3.0.CO;2-T

Table S3: Plasmids used for capped mRNA synthesis.
Linear. Enzyme: restriction enzyme used for linearization. *Hs: Homo sapiens*. * refers to morpholinos non-targeted sequences.

mRNA	Linear. Enzyme	Plasmids
ads1.L	*Xho*I	IRBHp990H0610D
*ads1.L**	*Xho*I	pBF-*adsl.L** from IRBHp990H0610D
Hs ADSL	*Not*I	pCS2^{+}-HsADSL
ppat.L	*Xba*I	IRBHp990H1017D
*ppat.L**	*Sal*I	pBF-*ppat.L** from IRBHp990H1017D
ppat.S	*Hind*III	IMAGp998O0914583Q
*ppat.S**	*Xho*I	pBF-*ppat.S** from IMAGp998O0914583Q
hprt1.L	*Not*I	IRBH990B02030D
*hprt1.L**	*Sac*I	pBF-*hprt1.L** from IRBH990B02030D
LacZ	*Xho*I	pSP6nucβgal

Table S4: Ribonucleotides probes used for *in situ* hybridization.
CDS: coding sequence. Linear. Enzyme : restriction enzyme used for linearization. pSK: p-BlueScript plasmid. RNA Pol.: RNA polymerase. UTR: untranslated region

Targeted genes	Probe	Linear. Enzyme	RNA Pol.	Plasmids
adsl.L	sense	*Kpn*I	T7	p5265 *adsl.L*-pSK (CDS +3'UTR; 480 bp) from IRBHp990H0610D
	antisense	*Sac*I	T3	p5265 *adsl.L*-pSK (CDS +3'UTR; 480 bp) from IRBHp990H0610D
adss1.L *adss1.S*	sense	*Sac*I	T3	p5634 *adss1.L*-pSK (5'UTR + CDS; 444 bp) from IRBHp990C0135D
	antisense	*Sal*I	T7	p5634 *adss1.L*-pSK (5'UTR + CDS; 444 bp) from IRBHp990C0135D
adss2.L	sense	*SacI*	T3	p5428 *adss2.L*-pKS (5'UTR + CDS; 444 bp) from IRBHp990E047D
	antisense	SalI	T7	p5428 *adss2.L*-pKS (5'UTR + CDS; 444 bp) from IRBHp990E047D
aprt.L	sense	*Kpn*I	T7	p5435 *aprt.L*-pKS (CDS +3'UTR; 779 bp) from IRAKp961E17253Q
	antisense	*Eco*RV	T3	p5435 *aprt.L*-pKS (CDS +3'UTR; 779 bp) from IRAKp961E17253Q
atic.L *atic.S*	sense	*Xho*I	SP6	P4915 *atic.L*-pExpress1 (CDS +3'UTR; 1854 bp) from IRBHp990G1167D
	antisense	*Eco*RI	T7	P4915 *atic.L*- pExpress1 (CDS +3'UTR; 1854 bp) from IRBHp990G1167D
hprt.L	sense	*Ase*I	T7	p5454 *hprt.L*-pKS (5'UTR + CDS; 592 bp) from IRBH990B02030D
	antisense	*Sac*I	T3	p5454 *hprt.L*-pKS (5'UTR + CDS; 592 bp) from IRBH990B02030D
gart.L	sense	*Bam*HI	T3	p5426 *gart.L*-pKS (CDS +3'UTR ; 530 bp) from IRAKp961C16299Q
	antisense	*Eco*RV	T7	p5426 *gart.L*-pKS (CDS +3'UTR ; 530 bp) from IRAKp961C16299Q
ppat.L *ppat.S*	sense	*Not*I	T3	p5527 *ppat.L*-pSK (CDS ; 481 bp) from XL.29008
	antisense	*Eco*RV	T7	p5527 *ppat.L*-pSK (CDS ; 481 bp) from XL.29008

Table S5: Oligonucleotides used for RT-PCR analyses. Amplification temperature and number of RT-PCR cycles were determined to obtain a single PCR product.

Amplified gene	Sense oligonucleotide (5'-3')	Antisense oligonucleotide (5'-3')	Amplification Temperature (°C)	Nb of cycles
ppat.L	GTTAGTCCCTGTGGCCGCT	GCCCATGCCCTTGTGCATTC	54	28
ppat.S	GAAGCGCGAGGTGTGTGTG	GCCCATGCCCTTGTGCATTC	58	29
gart.L	CAGAGACAGTTCTAGTGATTGG	GATAATCCCTGCTGCCAGAG	56	29
pfas.S	CTGACAGAACGTGCAGGG	CTAGTCTGGAACTTTATGAG	53	29
paics.1.S	CAGAACCACGTGGTACTGCC	GATCCCAGCCTCCTGCAGC	56	30
paics.1.L	GAAATGGAGTCTTACGCAGAAC	GATCCCAGCCTCCTGCAGC	53	27
paics.2.L	GCATTTGTTAAGAGGTGCAG	CCAAACTTAATCCTCATGTCC	55	28
adsl.L	ATGGCCTTCAACTTCAGCGA	AACGTTGGCATCTCTGCGTA	55	28
atic.L	GCTGCTAGCGACTTTATCCAG	GGGTACAGGTTACACACAAC	57	31
atic.S	GCTGTTTGCGGAGATGGAG	GGGTACAGGTTACACACAAC	53	30
adss1.L	CAAGTGGCATTATCAACCCC	CTTTGGATGAATATGTTGGTC	51	30
adss1.S	GTGGCATTATAAATCCTAAAGC	CTTTGGATGAATATGTTGGTC	53	29
Adss2.L	CCGCTACAGTAAGCGTAAC	GCTTTCATAGGAAATGGTGTG	51	28
hprt1.L	CTAAACATTATGCAGCCGATC	CATTCTTGCCTGTCAAGGTGG	54	26
hprt1.S	CTAAACACTACGCCGCCAGC	CATTCTTGCCTGTCAAGGTGG	52	29
aprt.L	CAGATTATGTCCGATCAGGAG	GCTAACAGATTCTGTGGGAC	57	29
gmps.L	GAGCGATGGGCAGAGATC	CTGTAAGAGCGACAGTCC	56	28
gmps.S	GAGGGTTGGGTAGAGAAC	CTGTAAGAGCGACAGTCC	53	29
odc1.L	GTCAATGATGGAGTGTATGGATC	TCCATTCCGCTCTCCTGACCAC	55	23

Table S6: Comparison of the purine biosynthesis pathways encoding genes and proteins between, *X. laevis*, *H. sapiens* and *X. tropicalis*. Alignments were performed using https://blast.ncbi.nlm.nih.gov. * XB: xenbase: https://www.xenbase.org/entry/

Xenopus laevis			*Homo sapiens*				*Xenopus tropicalis*				
Protein name	Accession Number	XB* Gene ID	Protein name	Accession Number	Identity %	Coverage %	Protein name	Accession Number	Identity %	Coverage %	Enzymatic activity
Ada.L<br>Ada.S	XP_018090401.1<br>NP_001085740.1	17336388<br>950506	ADA	NP_000013.2	74<br>70	97<br>98	Ada	NP_001011025.1	97	100	Adenosine deaminase
Ada2.L<br>Ada2.S	NP_001090531.1<br>NP_001089165	6254244<br>6251688	ADA2<br>ADA2	NP_001269154.1	57<br>59	96<br>97	Ada2<br>Ada2	XP_031754454.1	90<br>88	99<br>100	Adenosine deaminase
Ada.2.S	NP_001087740.1	5929096	ADA.2	No significant homolog			Ada.2	NP_001107369.1	90	100	Adenosine deaminase
Adk.S	NP_001086357.1	997231	ADK	NP_001114.2	82	95	Adk	NP_001016698	98	99	Adenosine kinase
Adsl.L	NP_001080593	380285	ADSL	NP_000017.1	83	93	Adsl	NP_001005457.1	96	100	Adenylosuccinate lyase
Adssl1.L<br>Adssl1.S	NP_001090012.1<br>NP_001087505.1	5758296<br>6254009	ADSS1<br>ADSS1	NP_689541.1	88<br>87	100<br>100	Adssl1<br>Adssl1	NP_001004939.1	97<br>96	100<br>100	Adenylosuccinate synthase
Adss2.L	NP_001080088.1	944129	ADSS2	NP_001117.2	86	94	Adss2	NP_989047.1	95	100	Adenylosuccinate synthase
Ak1.L<br>Ak1.S	NP_001087683.1<br>NP_001085451.1	6253770<br>6251725	AK1<br>AK1	NP_001305051.1	74<br>81	98<br>98	Ak1<br>Ak1	NP_001006817.1	91<br>100	97<br>100	Adenylate kinase
Ak2.L<br>Ak2.S	XP_018102289.1<br>NP_001080232.1	17344313<br>6254479	AK2<br>AK2	NP_001616.1	82<br>79	100<br>100	Ak2<br>Ak2	XP_012812152.1	96<br>97	100<br>100	Adenylate kinase
Ak3.L<br>Ak3.S	NP_001089446.1<br>NP_001084561.1	977031<br>17332462	AK3<br>AK3	NP_057366.2	74<br>75	97<br>97	Ak3<br>Ak3	XP_012812420.1	90<br>92	100<br>100	Adenylate kinase
Ak4.L<br>Ak4.S	XP_018113718.1<br>XP_018116248.1	17341782<br>959957	AK4	NP_001005353.1	77<br>74	96<br>94	Ak4<br>Ak4	XP_002931643.1	96<br>97	100<br>100	Adenylate kinase
Ak5.L<br>Ak5.S	XP_018113675.1<br>XP_018116212.1	6487811<br>17345311	AK5<br>AK5	AAH36666.1<br>NP_777283.1	72<br>71	100<br>100	Ak5<br>Ak5	XP_012815974.2	93<br>92	100<br>100	Adenylate kinase
Ak6.L<br>Ak6.S	NP_001089528.1<br>NP_001087040.1	972297<br>17335043	AK6<br>AK6	NP_057367.1	76<br>78	100<br>100	Ak6<br>Ak6	NP_001017167.1	95<br>97	100<br>100	Adenylate kinase

**Ak7.S**	NP_001081046.1	953552	AK7	NP_689540.2	63	99	Ak7	NP_001011352.1	93	100	Adenylate kinase
**Ak8.L**	NP_001088862.1	5831351	AK8	NP_689785.1	55	98	Ak8	NP_989104.1	90	100	Adenylate kinase
**Ak9.L**	XP_018118807	17335922	AK9	NP_001316531.1	52	34	Ak9	XP_031757751.1	85	96	Adenylate kinase
**Ak9.S**	XP_018118808	17335923	AK9		52	34	Ak9		85	96	
**Ampd1.L**	XP_018101841.1|	17335636	AMPD1	NP_000027.2	74	99	Ampd1	XP_002935821.2	95	100	Adenosine monophosphate deaminase
**Ampd1.S**	XP_018104675.1	17335637	AMPD1		72	99	Ampd1		92	100	
**Ampd2.S**	XP_018104829	6489067	AMPD2	NP_001355738.1	80	96	Ampd2	XP_031752724.1	96	100	Adenosine monophosphate deaminase
**Ampd3.L**	XP_018112762.1	6489056	AMPD3	NP_000471.1	80	99	Ampd3	NP_001025687.1	94	100	Adenosine monophosphate deaminase
**Ampd3.S**	XP_018115800.1	17344991	AMPD3		80	99	Ampd3		95	100	
**Aprt.L**	XP_018096991.1	6253243	APRT	NP_000476.1	65	100	Aprt	NP_001007941.1	93	100	Adenine phosphoribosyltransferase
**Atic.L**	NP_001090100.1	998812	ATIC	NP_004035.2	78	99	Atic	NP_001005460.1	95	100	Amino-Imidazole CarboxAmide Ribonucleotide transformylase and IMP cyclohydrolase
**Atic.S**	XP_018094436.1	17331460	ATIC		79	99	Atic		96	100	
**Gda.L**	NP_001083074.1	987047	GAH	NP_004284.1	61	93	Gda	XP_004910825.1	93	96	Guanine deaminase
**Gda.S**	XP_018099342.1	17337222	GAH		60	99	Gda		83	99	
**Gart.L**	NP_001093352.1	6251947	GART	NP_000810.1	75	98	Gart	XP_012813454.1	92	98	Phosphoribosylglycinamide-formyltransferase, phosphoribosylglycinamide synthetase and phosphoribosylaminoimidazole synthetase
**Gmps.L**	XP_018119259.1	950699	GMPS	NP_003866.1	92	98	Gmps	XP_002933290.3	97	98	Guanine monophosphate synthase
**Gmps.S**	XP_018121231.1	17340569	GMPS		93	100	Gmps		98	100	
**Guk1.L**	NP_001087146.1	6254574	GUK	NP_000849.1	70	97	Guk1	NP_001034818.1	89	100	Guanylate kinase
**Guk1.S**	NP_001086807.1	17334557	GUK		69	97	Guk1		90	100	
**Hprt1.L**	NP_001090235.1	6078713	HGPRT	NP_000185.1	89	99	Hprt1	NP_989312.1	100	100	Hypoxanthine-Guanine phosphorybosyl transferase
**Impdh1.L**	NP_001080792	957455	IMPDH1	NP_000874.2	92	100	Impdh1	NP_001017283.1	98	100	Inosine monophosphate dehydrogenase
**Impdh2.L**	NP_001083990.1	17345296	IMPDH2	NP_000875.2	93	100	Impdh2	NP_001008066.1	98	100	Inosine monophosphate dehydrogenase
**Impdh2.S**	NP_001082410.1	478732	IMPDH2		93	100	Impdh2		97	100	
**Paics.1.L**	NP_001080163	17334793	PAICS	NP_001072992.1	81	100	Paics.1	XP_012811124.2	96	100	Phosphoribosylaminoimidazole carboxylase Phosphoribosylaminoimidazolesuccino-carboxamide synthetase
**Paics.1.S**	NP_001086248.1	965998	PAICS		82	98	Paics.1		95	100	

**Paics.2.L**	XP_018085590.1	5872998	PAICS	NP_001072992.1	57	37	Paics.2	NP_001090685.1	86	97	Phosphoribosylaminoimidazole carboxylase<br>Phosphoribosylaminoimidazolesuccino-carboxamide synthetase
**Pfas.S**	XP_018094887.1	6485675	PFAS	NP_036525.1	65	99	Pfas	XP_012825646.2	93	99	Phosphoribosylformylglycinamidine synthase
**Pnp.L**<br>**Pnp.S**	NP_001079809.1<br>XP_018099448.1	1011603<br>17337633	PNP<br>PNP	NP_000261.2	68<br>66	95<br>93	Pnp<br>Pnp	NP_001006720.1	88<br>90	98<br>100	Purine nucleoside phosphorylase
**Ppat.L**<br>**Ppat.S**	NP_001083491.1<br>XP_041435416	972209<br>17340761	GPAT<br>GPAT	NP_002694.3	80<br>79	100<br>99	Ppat<br>Ppat	NP_989313.1	96<br>96	100<br>100	Phosphoribosylpyrophosphate amidotransferase
**Xdh.L**<br>**Xdh.S**	XP_018117710.1<br>XP_018120131.1	6485774<br>17331587	XDH<br>XDH	NP_000370.2	70<br>70	99<br>92	Xdh<br>Xdh	XP_031758260.1	89<br>90	100<br>100	Xanthine dehydrogenase/oxidase

Table S7: Comparison of the purine biosynthesis pathways encoding genes and proteins between *X. laevis* and *S. cerevisiae*. Alignments were performed using https://blast.ncbi.nlm.nih.gov. * XB: xenbase: https://www.xenbase.org/entry/.
[a] No significant alignment between the yeast and *X. Laevis* Hprt1.L entire sequences.
ada: adenine deaminase; xdh: xanthine dehydrogenase/oxidase

Xenopus laevis			***Saccharomyces cerevisiae***					
**Protein name**	**Accession Number**	**XB* Gene ID**	**Protein name**	**Accession Number**	**Identity %**	**SGD Locus ID**	**Coverage %**	**Enzymatic activity**
Ada.L<br>Ada.S	XP_018090401.1<br>NP_001085740.1	17336388<br>950506		No Ada activity in S.c.				Adenosine deaminase
Ada2.L<br>Ada2.S	NP_001090531.1<br>NP_001089165	6254244<br>6251688		No Ada activity in S.c.				Adenosine deaminase
Ada.2.S	NP_001087740.1	5929096	ADA.2	No Ada activity in S.c.				Adenosine deaminase
Adk.S	NP_001086357.1	997231	Ado1	NP_012639.1	40	*YJR105W*	91	Adenosine kinase
Adsl.L	NP_001080593	380285	Ade13	NP_013463.1	64	*YLR359W*	92	Adenylosuccinate lyase
Adssl1.L<br>Adssl1.S	NP_001090012.1<br>NP_001087505.1	5758296<br>6254009	Ade12	NP_014179.1	57<br>57	*YNL220W*	92<br>92	Adenylosuccinate synthase
Adss2.L	NP_001080088.1	944129	Ade12	NP_014179.1	56	*YNL220W*	92	Adenylosuccinate synthase
Ak1.L<br>Ak1.S	NP_001087683.1<br>NP_001085451.1	6253770<br>6251725	Adk1	NP_010512.1	28<br>28	*YDR226W*	84<br>92	Adenylate kinase
Ak2.L<br>Ak2.S	XP_018102289.1<br>NP_001080232.1	17344313<br>6254479	Adk1	NP_010512.1	55<br>59	*YDR226W*	94<br>90	Adenylate kinase
Ak3.L<br>Ak3.S	NP_001089446.1<br>NP_001084561.1	977031<br>17332462	Adk2	NP_011097.3	46<br>45	*YER170W*	86<br>86	Adenylate kinase
Ak4.L	XP_018113718.1	17341782	Adk2	NP_011097.3	43	*YER170W*	83	Adenylate kinase

Ak4.S	XP_018116248.1	959957			46		83	
Ak5.L<br>Ak5.S	XP_018113675.1<br>XP_018116212.1	6487811<br>17345311	Adk2	NP_011097.3	27<br>25	*YER170W*	61<br>58	Adenylate kinase
Ak6.L<br>Ak6.S	NP_001089528.1<br>NP_001087040.1	972297<br>17335043		none				Adenylate kinase
Ak7.S	NP_001081046.1	953552	Adk1	NP_010512.1	29	*YDR226W*	16	Adenylate kinase
Ak8.L	NP_001088862.1	5831351		none				Adenylate kinase
Ak9.L<br>Ak9.S	XP_018118807<br>XP_018118808	17335922<br>17335923	Adk1	NP_010512.1	24<br>24	*YDR226W*	14<br>14	Adenylate kinase
Ampd1.L<br>Ampd1.S	XP_018101841.1| XP_018104675.1	17335636<br>17335637	Amd1	NP_013677.1	51<br>51	*YML035C*	68<br>68	Adenosine monophosphate deaminase
Ampd2.S	XP_018104829	6489067	Amd1	NP_013677.1	53	*YML035C*	82	Adenosine monophosphate deaminase
Ampd3.L<br>Ampd3.S	XP_018112762.1<br>XP_018115800.1|	6489056<br>17344991	Amd1	NP_013677.1	55<br>48	*YML035C*	64<br>80	Adenosine monophosphate deaminase
Aprt.L	XP_018096991.1	6253243	Apt1	NP_013690.1	47	*YML022W*	93	Adenine phosphoribosyltransferase
Atic.L<br>Atic.S	NP_001090100.1<br>XP_018094436.1	998812<br>17331460	Ade17<br>Ade16<br>Ade17<br>Ade16	NP_013839.1<br>NP_013128.1<br>NP_013839.1<br>NP_013128.1	62<br>62<br>61<br>62	*YMR120C*<br>*YLR028C*<br>*YMR120C*<br>*YLR028C*	99<br>99<br>99<br>99	Amino-Imidazole CarboxAmide Ribonucleotide transformylase and IMP cyclohydrolase
Gda.L<br>Gda.S	NP_001083074.1<br>XP_018099342.1	987047<br>17337222	Gud1	NP_010043.1	39<br>39	*YDL238C*	96<br>91	Guanine deamnase
Gart.L	NP_001093352.1	6251947	Ade5,7<br>Ade8	NP_011280.1<br>NP_010696.3	46<br>29	*YGL234W*<br>*YDR408C*	96<br>31	Phosphoribosylglycinamide formyltransferase, phosphoribosylglycinamide synthetase and phosphoribosylaminoimidazole synthetase
Gmps.L<br>Gmps.S	XP_018119259.1<br>XP_018121231.1	950699<br>17340569	Gua1	NP_013944.1<br>NP_013944.1	37<br>36	*YMR217W*	93<br>94	Guanine monophosphate synthase

Guk1.L<br>Guk1.S	NP_001087146.1<br>NP_001086807.1	6254574<br>17334557	Guk1	NP_010742.1	55<br>55	*YDR454C*	92<br>92	Guanylate kinase
Hprt1.L	NP_001090235.1	6078713	Hpt1	NP_010687.3	34[a]	*YDR399W*	20[a]	Hypoxanthine-Guanine phosphorybosyl transferase
Impdh1.L	NP_001080792	957455	Imd2<br>Imd3<br>Imd4	NP_012088.3<br>NP_013536.3<br>NP_013656.1	60<br>63<br>63	*YHR216W*<br>*YLR432W*<br>*YML056C*	97<br>97<br>97	Inosine monophosphate dehydrogenase
IImpdh2.L<br>IImpdh2.S	NP_001083990.1<br>NP_001082410.1	17345296<br>478732	Imd2<br>Imd3<br>Imd4<br>Imd2<br>Imd3<br>Imd4	NP_012088.3<br>NP_013536.3<br>NP_013656.1<br>NP_012088.3<br>NP_013536.3<br>NP_013656.1	61<br>64<br>63<br>62<br>64<br>63	*YHR216W*<br>*YLR432W*<br>*YML056C*<br>*YHR216W*<br>*YLR432W*<br>*YML056C*	96<br>96<br>98<br>96<br>96<br>97	Inosine monophosphate dehydrogenase
Paics.1.L<br>Paics.1.S	NP_001080163<br>NP_001086248.1	17334793<br>965998	Ade1<br>Ade2<br>Ade1	NP_009409.1<br>NP_014771.3<br>NP_009409.1	25<br>31<br>26	*YAR015W*<br>*YOR128C*<br>*YAR015W*	51<br>29<br>51	Phosphoribosylaminoimidazole carboxylase<br>phosphoribosylaminoimidazolesuccinocarboxamide synthetase
Paics.2.L	XP_018085590.1	5872998	Ade2	NP_014771.3	31	*YOR128C*	14	Phosphoribosylaminoimidazole carboxylase<br>phosphoribosylaminoimidazolesuccinocarboxamide synthetase
Pfas.S	XP_018094887.1	6485675	Ade6	NP_011575.1	37	*YGR061C*	93	Phosphoribosylformylglycinamidine synthase
Pnp.L<br>Pnp.S	NP_001079809.1X<br>P_018099448.1	1011603<br>17337633	Pnp1	NP_013310.1	52<br>45	*YLR209C*	87<br>91	Purine nucleoside phosphorylase
Ppat.L<br>Ppat.S	NP_001083491.1<br>XP_041435416	972209<br>17340761	Ade4	NP_014029.1	36<br>36	*YMR300C*	91<br>92	Phosphoribosylpyrophosphate amidotransferase
Xdh.L<br>Xdh.S	XP_018117710.1<br>XP_018120131.1	6485774<br>17331587		No Xdh activity in *S.cerevisiae*.				Xanthine dehydrogenase/oxidase
